# The Present and Future of Insect Biodiversity Conservation in the Neotropics: Policy Gaps and Recommendations

**DOI:** 10.1007/s13744-023-01031-7

**Published:** 2023-03-14

**Authors:** Natalie E. Duffus, Alejandra Echeverri, Lena Dempewolf, Jorge Ari Noriega, Paul R. Furumo, Juliano Morimoto

**Affiliations:** 1 Doctoral Training Centre, Univ of Oxford, Oxford, UK; 2 Centre for Conservation Biology, Dept of Biology, Stanford Univ, CA Stanford, USA; 3 The Natural Capital Project, Stanford Univ, CA Stanford, USA; 4 Ministry of Planning and Development, Government of the Republic of Trinidad and Tobago, Caribbean, Trinidad and Tobago; 5 Grupo Agua, Salud y Ambiente, Facultad de Ingeniería, Universidad El Bosque, Bogotá, Colombia; 6 Stanford Doerr School of Sustainability, Stanford Univ, Stanford, USA; 7 School of Biological Sciences, Univ of Aberdeen, Aberdeen, Scotland; 8 Programa de Pós-Graduação Em Ecologia E Conservação, Univ Federal Do Paraná, Curitiba, Brazil; 9 Institute of Mathematics, Univ of Aberdeen, King’s College, Aberdeen, Scotland

**Keywords:** Environmental policy, Entomology, Neotropical biodiversity, Insect declines

## Abstract

Emerging evidence suggests that insect populations may be declining at local and global scales, threatening the sustainability of the ecosystem services that insects provide. Insect declines are of particular concern in the Neotropics, which holds several of the world’s hotspots of insect endemism and diversity. Conservation policies are one way to prevent and mitigate insect declines, yet these policies are usually biased toward vertebrate species. Here, we outline some key policy instruments for biodiversity conservation in the Neotropics and discuss their potential contribution and shortcomings for insect biodiversity conservation. These include species-specific action policies, protected areas and Indigenous and Community Conserved Areas (ICCAs), sectoral policies, biodiversity offsetting, market-based mechanisms, and the international policy instruments that underpin these efforts. We highlight that although these policies can potentially benefit insect biodiversity indirectly, there are avenues in which we could better incorporate the specific needs of insects into policy to mitigate the declines mentioned above. We propose several areas of improvement. Firstly, evaluating the extinction risk of more Neotropical insects to better target at-risk species with species-specific policies and conserve their habitats within area-based interventions. Secondly, alternative pest control methods and enhanced monitoring of insects in a range of land-based production sectors. Thirdly, incorporating measurable and achievable insect conservation targets into international policies and conventions. Finally, we emphasise the important roles of community engagement and enhanced public awareness in achieving these improvements to insect conservation policies.

## Introduction

Insects are responsible for a wide range of functional roles within the ecosystem, contributing to the ecosystem service framework (Metcalfe et al. 2014; Noriega et al. 2018; Ollerton 2021; Seibold et al. 2021). These include conventional services such as pollination (Klein et al. 2007; Gallai et al. 2009; Klatt et al. 2014) and dung degradation which maintains soil fertility and controls pests (Nichols et al. 2008). Furthermore, insects provide a range of unconventional ecosystem services (Morimoto 2020), including plastic degradation (Bombelli et al. 2017) and numerous contributions to human culture and tourism (Duffus et al. 2021 and Jacinto-Padilla et al. 2021). These insect ecosystem services are critical for achieving the UN Sustainable Development Goals and long-term global sustainability (Dangles and Casas 2019). However, at present, anthropogenic modification of global landscapes is contributing to insect population decline (Seibold et al. 2019; Bowler 2021; Boyes et al. 2021), with reduced diversity posing a threat to the sustainability of ecosystem services provided by insects (Soliveres et al. 2016; Newbold et al. 2019; Noriega et al. 2021). Additionally, these declines compromise the Sustainable Development Goal 15, which sets out to conserve natural populations of species and prevent extinctions (United Nations 2015).

Changes to the diversity and abundance of insect populations have been identified in areas of the Northern Hemisphere, mainly in Europe, leading to concerns of similar patterns being observed on a global scale (Dirzo et al. 2014; Bell et al. 2020; Cardoso et al. 2020; Wagner 2020). In the Neotropics, however, there is very little data on the status of insect biodiversity, even though the region hosts essential habitats that are considered global biodiversity hotspots, including Brazil’s Cerrado and Atlantic forests, the Caribbean, Central Chile, and the Mesoamerican hotspot (Myers et al. 2000). One reason for the general lack of data on insect decline in the Neotropics is that biodiversity databases such as the Global Biodiversity Information Facility (GBIF) exhibit biases toward the Northern Hemisphere, with significant taxonomic and geographic gaps for the Southern Hemisphere, including the Neotropics (Rocha-Ortega et al. 2021).

In GBIF, only 5752 insect species from South America are included, as opposed to 25,050 insect species from North America (Rocha-Ortega et al. 2021). While taxonomic coverage can be reduced by incorporating more data from other sources, this can increase the prevalence of other biases, including temporal bias (Boyd et al. 2022). The lack of data in the region can obscure patterns of insect extinction that remain undetected and, thus, unmanageable from the perspective of conservation policies (Janzen and Hallwachs 2019, 2021). Existing data has indeed identified declines in the abundance and diversity of several insect taxa in the Neotropics, including Hymenopterans (Frankie et al. 2009; Nemésio 2013), Lepidopterans (Salcido et al. 2020), Hemipterans (Pinedo-Escatel et al. 2021), and aquatic insects (Romero et al. 2021). This includes, for example, a 53% decline in sap-sucking Hemiptera (tribe: Athysanini) over 75 years in Mexican dry forests (Pinedo-Escatel et al. 2021). Modelling efforts also indicate the potential for further losses in the face of climatic changes (Fonseca 2009; Giannini et al. 2012; Gonzalez et al. 2021), which may pose a threat to ecosystem services in the area, including pollination, with up to US$22 billion of crops in Latin America attributed to insect pollinators (Basualdo et al. 2022). Therefore, it is critical to address such declines, not just for the intrinsic value of insect species, but for the functions underpinning ecosystem stability to ensure the continuity of ecosystem services essential to society.

To undertake conservation action, priorities are often determined by tools such as the International Union for Conservation of Nature (IUCN) Red List. However, despite the Neotropics being estimated to hold a large proportion of global insect biodiversity (Stork 2018), only 2277 insect species feature on the IUCN Red List for the Neotropical biogeographical realm (IUCN 2022), of which 1.8% are experiencing population decline, 0.3% population growth, 8.6% are stable and the majority (87.5%) has an unknown population trend. However, even these trends are unlikely to represent the overall trends for insect biodiversity of the region, given that 79% of the Neotropical insects in the IUCN Red List belong to the group Odonata. In comparison, species-rich orders such as Coleoptera, Hymenoptera, and Diptera account for only 12.2%, 2.1%, and 0.04% of the insects listed.

Since the IUCN Red List is an easily recognisable tool for the public and policymakers, and can play a critical role in informing conservation decision-making (Rodrigues et al. 2006; Betts et al. 2020), species must be identified, described and monitored to determine long-term trends. The taxonomic skew in the IUCN Red List may reflect the lack of resources for taxonomy in the region and the small number of established specialist taxonomists for diverse, lesser-studied taxa in the Neotropics (Brown 2005; New and Samways 2014). This has knock-on effects on the inclusion of species in the IUCN Red List and the design of conservation policies. Undescribed and data-scarce insect species are not included, with the undesirable effect of underestimating the resources needed for insect conservation as opposed to vertebrate conservation (Donaldson et al. 2017; Davies et al. 2018; Mammola et al. 2020). This has biased policies toward vertebrates and well-studied insect groups (Cardoso 2012; Leandro et al. 2017; Duffus and Morimoto 2022).

With such limited data, there is a burgeoning need for frameworks, initiatives and policies that protect insect biodiversity and reduce extinction risks of insect biodiversity in the Neotropical region (Forister et al. 2019; Cardoso et al. 2020). Furthermore, there exists enough evidence of insect population decline (Frankie et al. 2009; Nemésio 2013; Salcido et al. 2020; Pinedo-Escatel et al. 2021; Romero et al. 2021; Lewinsohn et al. 2022) to warrant action now, to prevent further such declines, and ensure the continuity of essential ecosystem functions and services that insects perform. Here, we discuss existing policy instruments for conservation in the Neotropics, their potential to conserve insect populations indirectly, and future steps to enable more direct protection of insect populations.

## The Neotropical biodiversity conservation policy mix

There are many known threats to biodiversity globally, which include habitat loss and fragmentation (Newbold et al. 2015; Maxwell et al. 2016; Fletcher et al. 2018), pesticide use (Goulson 2013; Sluijs et al. 2013; Sánchez-Bayo 2014; Marques et al. 2020), invasive species (Englund 2008; Wagner and Driesche 2010; Tallamy et al. 2021), pollution (Grubisic et al. 2018; Feldhaar and Otti 2020; Boyes et al. 2021), and climate change (Halsch et al. 2021). These factors interact and reduce the quantity and quality of available habitat for insect populations, which ultimately contribute to subsequent insect decline and extinction (Côté et al. 2016; Ito et al. 2020; Raven and Wagner 2021; Bowler 2021). For example, the Calliphoridae (blowfly) species *Neta chilensis* (Walker, 1836) which may be extinct due to the interaction of multiple stressors (Mulieri et al. 2022). Therefore, policies that mitigate the synergistic effect of threats to biodiversity are essential for effective insect conservation. Below, we outline some examples of biodiversity conservation policies currently implemented in the Neotropical region, discuss their relevance to insect conservation goals, and areas where more directive steps could be taken.

### Traditional conservation policies

#### Species protection and prioritisation

One of the earliest nature conservation approaches to be developed was protected species laws, which were typically created to preserve mammal populations that were overexploited by hunters (Epstein 2006). Regulations for the conservation of listed species continue to be common in places such as Europe, albeit with a persistent bias toward vertebrates to the detriment of invertebrate groups (Cardoso 2012; Leandro et al. 2017; Duffus and Morimoto 2022), a bias that cannot be justified by differences in extinction risk (Moser et al. 2016). One such example from the Neotropics is the Environmental Management Act 2000 in Trinidad and Tobago (Ministry of Legal Affairs 2009). This act denotes “Environmentally Sensitive Species” (ESSs), which are resident in Trinidad and Tobago, and is in danger of extinction. The act can prohibit the killing, collecting or disturbance of the ESSs. However, only ten species are listed as ESSs, all of which are vertebrate species (Government of Trinidad and Tobago 2022). Another instance is the General Wildlife Law in Mexico which sets out species at risk of extinction, for which the Secretariat will promote their conservation and protection (The General Congress of the United Mexican States 2021). The current version of the list details 46 invertebrate species, of which just three are insects, versus 292 mammal species (The General Congress of the United Mexican States 2010).

A broader initiative exists in Brazil, in the creation of PAN’s (Plano de Ação Nacional), which seek to increase conservation action for threatened species, habitats and ecosystems (Chico Mendes Institute for Biodiversity Conservation 2022). Two of these plans have specifically targeted insects—the first being the “Plano de Ação Nacional para Conservação de Lepidópteros” which ran from 2010 to 2015 (Chico Mendes Institute for Biodiversity Conservation 2022). This plan included 8 goals and 76 actions to benefit Lepidopterans nationally (Chico Mendes Institute for Biodiversity Conservation 2011). These actions ranged from finding remnant populations of critically endangered species, to standardizing methods for monitoring, and even increasing resources for taxonomy, parataxonomy and publishing updated species lists (Chico Mendes Institute for Biodiversity Conservation 2011). Though this plan has expired, from 2023 the Plano de Ação Nacional para a Conservação dos Insetos Polinizadores is in effect, outlining 71 actions for the conservation of 56 pollinating bee and Lepidoptera species (Chico Mendes Institute for Biodiversity Conservation 2022). This focus on Lepidopterans follows the bias in policies in the UK (Duffus and Morimoto 2022), suggesting that Lepidopterans, and pollinating insects more broadly, potentially have higher perceived value for policymakers. Having said that, the efforts to protect insects (in Brazil and elsewhere) should ideally be extended to other functional groups, such as decomposers, parasitoid and saxoprylic insects, which fulfil key roles in the ecosystem but are less well studied (Nichols et al. 2008; Ramos et al. 2020; Seibold et al. 2021; Shaw and Hochberg 2001).

There is an argument that the designation of a handful of species as a conservation priority can offer conservation to other species with similar habitat requirements. These species act as “umbrella” species for others (Spitzer et al. 2009; Branton and Richardson 2011). For example, the umbrella utility of the Jaguar (*Panthera onca* (L., 1758)) conservation network from Mexico to Argentina for other mammals has been demonstrated (Thornton et al. 2016). However, concerns about the broader effectiveness of the umbrella species approach (Simberloff 1998; Roberge and Angelstam 2004) must be considered when designing conservation efforts in the Neotropics. For instance, the conservation of representatives from higher taxa (*e.g.,* mammals) does not necessarily ensure the preservation of other taxa (Roberge and Angelstam 2004). This is particularly true where species umbrellas do not directly address the specific threats to a group, such as tourism in cave microhabitats (Pacheco et al. 2021) or pesticide use (Sánchez-Bayo 2014). It also must be recognised that insects themselves can work as umbrella species (Pérez-Espona 2021; Fierro and Vergara 2019; Whiteman and Sites 2008), although in practice this may not occur owing to their lack of “flagship” characteristics (Simberloff 1998). Nonetheless, the utility of insects as umbrella species cannot be overlooked, particularly where insects do not co-occur with charismatic vertebrate umbrellas (Whiteman and Sites 2008).

To fully ascertain the efficacy of policies targeting species, we should identify species that may be endangered by using long-term standardised population monitoring data and adequate estimation of population size to generate a robust evaluation of extinction risk (Hambler and Henderson 2019; Montgomery et al. 2020; Didham et al. 2020). This robustness is vital because IUCN Red List criteria for species status assessments can produce inconsistent insect assessments (Fox et al. 2019) and thus require increased objectivity and standardisation (Cardoso et al. 2011b; Collen et al. 2016). In addition, using new and emerging technologies could present a cost-effective way to generate baseline data in the Neotropics, including acoustic monitoring techniques (Aide et al. 2013; Deichmann et al. 2018).

Moreover, when considering extinction risk assessments for conservation, it must be recognised that the Neotropical region possesses high levels of insect endemism (Löwenberg-Neto and Carvalho 2009). The extinction risks of endemic insects are more readily recognised by country-specific local red lists, rather than the global IUCN Red List, with 3.4 × more endemic insect assessments on local red lists than the IUCN Red List (Barahona-Segovia and Zúñiga-Reinoso 2021). Integrating local red list assessments into the overarching IUCN Red List could increase recognition of the conservation need of such endemic insects, fuelling increased funding for research to inform species-specific policies (Barahona-Segovia and Zúñiga-Reinoso 2021). This would raise the plight of insect species in need of conservation to policymakers, increasing their representation on policies, such as those denoting species of conservation priority in Trinidad and Tobago and Mexico.

#### Area-based interventions: protected areas and Indigenous Community Conserved Areas

Land protection has been considered a more effective use of resources than species protection laws, given the significant taxonomic gaps in our data for Neotropical insects (Lewinsohn et al. 2005). Areas designated as protected are set aside for biodiversity conservation, education or tourism, with reduced (or no) scope for economic activities such as agriculture and forestry. Such areas include national parks, wilderness areas and strict nature reserves as defined by the IUCN, all with differing levels of anthropogenic impact permitted (Dudley 2013). Thus, protected areas have benefits for society by reducing poverty, securing employment opportunities and providing many health benefits (Naidoo et al. 2019; Ma et al. 2020), and have been regarded as one of the most important use of funds for insect conservation globally (Miličić et al. 2021).

The planning of protected areas typically employs modelling to determine sites of high species diversity, or sites considered vulnerable and irreplaceable (Margules et al. 2002; Mokany et al. 2014). However, policies that create protected areas can fail to encapsulate areas of vital insect habitat (Powell et al. 2000; Rodriguex-Cabal et al. 2008; Megna et al. 2021). For example, models of existing networks have been shown to exclude endemic species, such as three endemic Dytiscidae beetles in Cuba’s National Protected Area’s System (Megna et al. 2021). Additionally, some of the diverse ecoregions found in the Neotropics can be underrepresented by protected area networks (Hazen and Anthamatten 2004; Soutullo and Gudynas 2006; Cantú-Salazar and Gaston 2010; Durán et al. 2013). This includes temperate grasslands, deserts and xeric shrublands, the latter of which can hold unique insect faunas, such as in the Atacama (Zúñiga-Reinoso and Predel 2019; Pizarro-Araya et al. 2021). The exclusion of these biomes from protected area networks leave their biodiversity vulnerable to anthropogenic pressures. The underrepresentation of ecoregions in protected area networks could be driven by sampling biases, with areas including the Caatinga and Pantanal being less intensively sampled than other biomes, such as forest (Lewinsohm et al. 2005; Oliveira et al. 2016; Ramos et al. 2020; Silva et al. 2017). Moreover, even protected areas can even be misplaced within biodiversity hotspots. For instance, in the Tropical Andes, an area of high endemism (Löwenberg-Neto and Carvalho 2009; Särkinen et al. 2012), 77% of protected areas fall in areas of low conservation priority (Bax and Francesconi 2019). Moving forward, this highlights the need to ensure protected area networks represent all ecoregions and conservation priorities.

Alongside protected areas, there are many Indigenous and ethnic territories in the Neotropics, which provide a different area for the conservation of insect biodiversity. These Indigenous and Community Conservation Areas (ICCAs) are highly effective in preventing encroaching land expansion (Schwartzman and Zimmerman 2005; Carranza et al. 2014; Paiva et al. 2015). Many Indigenous People’s and Local Communities (IPLCs) have the right to free, prior and informed consent on all administrative and legislative measures, as well as public and private projects, which involve their territories (Bonilla-Mejía and Higuera-Mendieta 2019). Thus, working with IPLCs on insect conservation projects is essential to guarantee the persistence of many insect species. These efforts should be prioritised for several reasons. Firstly, ICCAs comprise a large portion of the land across the Neotropics, home to many endemic insect species (Fletcher et al. 2021). In some instances, ICCAs can hold similar—or greater—levels of animal diversity than comparable protected areas (Schuster et al. 2019). Secondly, these areas can be more effective than state-designated protected areas at conserving biodiversity and preventing deforestation under certain circumstances (Jonas 2017; Bonilla-Mejía and Higuera-Mendieta 2019). Thirdly, Traditional Ecological Knowledge (TEK) of different IPLCs can aid the conservation of declining species. In India, engaging with farmers has provided evidence of declines in pollinating insects, for which no data was previously available (Smith et al. 2017). Another example of utilising TEK could be working with IPLCs in Latin America, where several species of Orthoptera constitute part of the diet, from this we can learn local knowledge on species habitats and behaviour that could aid in their conservation (Melo-Ruiz et al. 2011). These intercultural approaches that focus on co-creating knowledge and conservation practices between IPLCs and conservation biologists, and in some cases, Indigenous biologists, inside ICCAs can be augmented by enhancing insect distribution modelling to identify areas of priority insect habitat.

### Sectoral policies

#### Agriculture

Agriculture and cattle ranching are key drivers of insect decline in the Neotropics through habitat loss, degradation and agrochemical use (Fearnside 2005; Klink and Machado 2005; Freitas et al. 2009; Kehoe et al. 2017). These threats have been mainly addressed by sectoral policies that support more sustainable agricultural production by deterring agricultural land conversion and promoting wildlife-friendly management practices. In addition, agroecological systems like shade-grown coffee, silvopasture and other diversified farming systems support insect diversity by enhancing habitat connectivity and creating corridors for the movement of species between protected areas and ICCAs in the landscape (Wangchuk 2007; McDermott and Rodewald 2014; Gutiérrez-Chacón et al. 2020; Samways et al. 2020). These policies are numerous and varied (Kremen and Merenlender 2018), but primarily rely on voluntary standards and market-based strategies that target the trade of commodities like sugar cane, coffee, cacao, oil palm and dairy (Englund and Berndes 2015; Furumo and Lambin 2020).

Many sustainability standards regulate the use of certain agrochemicals and GMO crops, promote integrated pest management strategies and require management plans for endangered species found within production areas (Englund and Berndes 2015). This can potentially benefit insects in the Neotropics, given that many are at risk from pesticide and herbicide use (Abraham et al. 2018; Padilha et al. 2020; Battisti et al. 2021; Smith et al. 2021; Almeida et al. 2021). Nonetheless, some harmful pesticides are still permitted under such standards. For example, Rainforest Alliance-certified banana farms in Costa Rica have been found to have similar pesticide application practices to non-certified farms, and less insect community diversity than non-certified and organic farms (Bellamy et al. 2016; Beekman et al. 2019). The ecological pillar of certification standards typically prioritises the monitoring of habitat and vertebrates on farms, leaving gaps for insect conservation. Given the economic importance of the agricultural sector in Latin America, government regulations also fail to limit harmful pesticide use effectively. For instance, 9.6% of approved pesticides in Chile are banned in Europe for their detrimental effects on wildlife (Henríquez-Piskulich et al. 2021).

This presents the importance of policies in the agricultural sector that take more directive steps to reduce the use of pesticides which are significantly detrimental to native insect populations (Abraham et al. 2018; Padilha et al. 2020; Battisti et al. 2021; Smith et al. 2021; Almeida et al. 2021). Such steps are already being taken, for example, in Brazil, where the number of biocontrol-based solutions has greatly increased (Togni et al. 2019). However, further work needs to ensure that these products are compatible with organic agriculture (Togni et al., 2019) and that instruments such as the Nagoya Protocol do not slow the development of such solutions (Lenteren 2020). Furthermore, consumer countries continue to import food from this region, thus contributing to biodiversity loss within these biodiversity hotspots, outside of their borders (Wilting et al. 2017). Therefore, voluntary certifications targeting consumers aiming to make sustainable choices also have a role to play in ensuring certified farms minimise harmful pesticide use, but the agrochemicals permitted under these programmes should be revised to ensure that standards also protect insect communities (Bellamy et al. 2016; Beekman et al. 2019). Additionally, in Europe, there have been steps to ensure products in the EU market do not contribute to deforestation and degradation (European Commission 2022), representing a step forward in preventing consumerism outside the Neotropics from adversely affecting biodiversity there.

#### Forestry

Many Neotropical countries are highly forested and these ecosystems are experiencing rapid land conversion that threatens insect populations (Banerjee et al. 2021) (de Lima et al. 2020). Neotropical forests are governed by policies in three domains: national and subnational government policies, international REDD + (reducing emissions due to deforestation and forest degradation) financial mechanisms, and sustainable supply chain initiatives (Furumo and Lambin 2020). In compliance with the Paris Agreement, many Latin American and Caribbean countries leverage REDD + finance to halt domestic deforestation (Hein et al. 2018). While biodiversity conservation does not currently fall under REDD + , the initiatives have the potential to indirectly impact insect species by protecting their habitat. This is especially pertinent in light of evidence that particular practices such as low-impact selective logging can prevent irreplaceable impacts upon insect communities, such as dung beetles (de Moura et al. 2021).

The forestry sector’s primary sustainable supply chain initiative has been the Forest Stewardship Council (FSC) certification, a voluntary standard that large companies also use to demonstrate compliance under sustainable timber procurement pledges (*e.g*., IKEA). As in the case of agricultural standards, forestry standards like FSC forbid natural habitat conversion, request endangered species management plans in plantation areas and promote biodiversity-friendly practices including intercropping (Englund and Berndes 2015). Yet, the forestry sector still negatively impacts many insects (including natural predators) because of pesticide spraying to control timber pests such as leaf-cutting ants and termites (Zanuncio et al. 2016). While the FSC principles and criteria have a pesticide policy that requires the prevention of using highly hazardous pesticides, and promoting non-chemical methods for pest control, many plantation managers spray with chemical pesticides as early pest infestations can damage entire plantations (Zanuncio et al. 2016). Additionally, groups such as the Roundtable on Sustainable Palm Oil (RSPO) provide little guidance, suggesting that agrochemical use should be “minimised” (Furumo et al. 2019). More stringent pesticide regulation in forestry policy could prevent detrimental impacts on Neotropical insects, for instance, by banning pesticides which have been demonstrated to be harmful in other regions, such as Europe (Henríquez-Piskulich et al. 2021) or using the World Health Organisation classification of hazardous chemicals (WHO 2019).

### Biodiversity offsetting and ecological restoration

Biodiversity offsetting policies typically aim to ensure no net loss of biodiversity under development projects, by avoiding and minimising losses and harms, and offsetting any unavoidable losses (Arlidge et al. 2018). Biodiversity offsetting can be achieved by government policies or voluntary obligations from private corporations and lenders, with areas from the latter tending to be larger (Bull and Strange 2018). However, the voluntary nature of some schemes and increased flexibility in strategies have put the offsetting approach under scrutiny (Gordon et al. 2015; Guillet and Semal 2018; zu Ermgassen et al. 2020). Despite this, in 2018, Central and South America contain a very large area of offset projects, with 45% of the world’s offset area (Bull and Strange 2018). This is in addition to other restoration projects being undertaken, such as the Bonn Challenge, in which many Neotropical countries including Argentina, Brazil, Colombia, El Salvador, and Honduras, have each pledged to restore millions of hectares of degraded landscapes (Bonn Challenge 2022). Therefore, insect biodiversity has great potential to benefit from biodiversity offsetting and the restoration of degraded habitats, particularly through the restoration and management of habitats that are otherwise not included by protected area networks (*e.g*., temperate grasslands).

It is vital to ensure that biodiversity offsets do not assume functional equivalence of species and maintain the diversity of insect species which ensures the long-term provisioning of ecosystem services (Clavel et al. 2011; Plas et al. 2016). The strengths and weaknesses of individual offsetting and restoration policies and initiatives should be weighted before assuming their conservation value for insect biodiversity (McKenney and Kiesecker 2010; Grimm and Köppel 2019; Pope et al. 2021). Moreover, biodiversity assessments need to be undertaken in a way that values insect species, alongside other features of the habitat, such as vegetation.

### Market-based mechanisms

Payments for Ecosystem Services (PES) is another strategy that addresses the economic externalities of resource extraction and commodity production to improve social and ecological outcomes (Chan et al. 2017). They act as environmental incentive programmes, which involve users of ecosystem services paying for actions that protect those services (Chaplin-Kramer et al. 2019). PES programmes for aquatic habitats, known as water funds (Brauman et al. 2019), offer a policy instrument to protect aquatic insect biodiversity in addition to protected areas. Since 2006, more than 40 water funds have been established in Latin America (Guerry et al. 2015). These funds, established by cities, work as payments from downstream water consumers to upstream communities that can alter land management practices to improve water quality and quantity (Guerry et al. 2015). Despite the purpose of most PES schemes being to pay for services such as carbon storage and water provision, PES water schemes can protect aquatic insect biodiversity indirectly by funding land stewards to preserve natural habitats in private lands (Brauman et al. 2019). PES schemes can protect critical natural areas that serve as habitats for many species (Chan et al. 2017). Nonetheless, some critical ecosystems, including arid shrublands and deserts, are underrepresented by PES schemes (Wunder 2007). These ecosystems contain unique climate-sensitive insect communities (Tirado et al. 2018) that could benefit indirectly from such market-based incentives.

### International policy instruments

Finally, an essential facet of the policy mix to consider is international policy instruments for biodiversity conservation. This includes the Convention on Biological Diversity (CBD), a key purpose of this legally binding convention being biodiversity conservation (United Nations 1992). Recognising that countries in the Neotropics are parties of this convention, many of their domestic policies outlined above will be based upon the goals and objectives of the convention. The international-level interest in biodiversity stems from the challenges associated with the fact that many countries with the highest levels of biodiversity also have the fewest resources available to conserve it, e.g. in the Neotropics (Swanson 1999). However, targets set under this convention have largely been unachieved (Secretariat of the Convention on Biological Diversity 2020), owing to low resource input, and a lack of measurability to ensure compliance (Green et al. 2019; Xu et al. 2021).

Conventions such as the CBD also exhibit more specific limitations for insects. For example, reporting on progress toward the Aichi targets did not feature evaluations of changes in extinction risk of insect species (Secretariat of the Convention on Biological Diversity 2020). Moreover, current preparations of the post-2020 global biodiversity framework—which affect the agriculture, forestry, tourism, manufacturing, fisheries and oil and gas sectors—have a broad-scale focus on habitat loss and regulating living modified organisms, with little attention being paid to Neotropical insects (Convention on Biological Diversity 2020). This could be attributable to the fact that reporting on target progress focuses on trends from the IUCN Red List, data from which is limited to a number of insect taxa (IUCN 2022). The IUCN Red List tends to exclude species with small body sizes, narrow distribution ranges and low dispersal abilities, which constitute the vast majority of the Neotropical insects (Cardoso et al. 2011a). Therefore, a concentrated effort to evaluate the extinction risk of insect species in the Neotropics is required.

Additionally, another legally binding convention, the Convention of International Trade in Endangered Species of Wild Fauna and Flora (CITES) forms a large part of countries’ efforts toward reducing species extinction (CITES 1973). This convention focuses on species threatened by trade, with only 79 insects currently included, from the orders Lepidoptera and Coleoptera (CITES 1973). This includes just six insects from the Neotropics (CITES 1973), despite the illegal trafficking of butterflies bringing around US$200 million a year to the global economy (Speart 2012). Not only does the trade of insects increase their extinction risk (Crespin and Barahona-Sergovia, 2021), but it also can facilitate the spread of disease and invasive species (Carvalho 2022). This low representativity of insects on CITES means that if countries base their biodiversity policy upon CITES, they may be biased toward vertebrate species and fail to curb the trafficking of at-risk insects. Explicit incorporation of achievable and measurable insect population conservation targets into international policy instruments such as the CBD and CITES would ensure that countries recognise species’ conservation needs beyond vertebrates. Assessment of more insect species extinction risk on the IUCN Red List would underpin this, providing a resource for policymakers to base policy upon (see Sect. 2.1).

## Governance complexity

A common factor that dictates the success of direct and indirect policies is the socio-political landscape in which the policies are designed and implemented. A fundamental challenge for conservationists is garnering support for insect conservation when society can frequently be unaware of the diversity and importance of insects (Cardoso et al. 2011b). This challenge is amplified in the tropics where the immediate need for economic development opportunities may overshadow the less conspicuous threats of insect extinction. The success of the policies mentioned above hinges upon funding, appropriate law enforcement and political support, which are susceptible to political ideologies and corruption (Smith and Walpole 2005).

For example, broadly across the Neotropics, the downgrading and downsizing of protected areas to allow industrial activities means that protected areas cannot be relied upon as permanent entities for conservation (Mascia and Pailler 2011; Mascia et al. 2014; Watson et al. 2014). In most cases, downgrading and downsizing of protected areas are carried out in opposition to conservation objectives to accelerate economically profitable industrial activities. This jeopardises the ability of a state-designated protected area to safeguard insect habitats and thus conserve their populations (Mascia et al. 2014).

Firm governance can be linked to environmental concern, with political stability, accountability and transparency being important to favourable outcomes from conservation projects (Smith and Walpole 2005; Baynham-Herd et al. 2018). Unfortunately, areas of the Neotropics have lower levels of governance stability tied to lower levels of environmental concern and higher corruption (Baynham-Herd et al. 2018; Inter-American Development Bank 2020; Pinheiro et al. 2020). Furthermore, political ideologies also influence the ecological concern of governments. This is exemplified in Brazil, where existing environmental legislation is currently being dismantled, presenting a threat to biodiversity conservation (Abessa et al. 2019; Ferrante and Fearnside 2019).

Environmental concerns can be raised among the general population, which can be a tool to influence policymakers to incorporate insects into policy. This begins with creating more positive perceptions of insects by increasing public knowledge of insects and “marketing” their value (Wilson et al. 2004; Hart and Sumner 2020). The IUCN Red List is one such tool for this (Rodrigues et al. 2006), and given the general public preference for endemism (Meuser et al. 2009), national red lists present an opportunity to educate people on the plight of endangered endemic species in Neotropical countries (Barahona-Segovia and Zúñiga-Reinoso 2021). Museums and natural history collections also offer an opportunity for education; however, these are subject to a lack of funding and support (Suarez and Tsutsui 2004; Norris 2017).

Additionally, community engagement is an effective tool and examples of policies that foster greater environmental awareness through conservation engagement already exist. For instance, in the Colombian Amazon, beekeeping of native stingless bees is encouraged (Gonzalez et al. 2021). With over 28% of Colombia’s stingless bee species being used in beekeeping (Nates-Parra and Rosso-Londono 2013), this is an opportunity for increased environmental awareness and conservation as well as improvements to human-well-being and poverty alleviation (Chanthayod et al. 2017). However, these policies must be implemented along with controls to prevent the spread of species and diseases out of their native range, which is currently lacking (Gonzalez et al. 2021). This could be akin to the Beekeeping and Bee Products Act from Trinidad and Tobago, which protects native stingless Meliponini bee species from mismanaged honeybees (*Apis mellifera* (L.,1758)) (Ministry of Legal Affairs 2013). Another tool which can successfully raise awareness of insects (and invertebrates more widely) and promote their conservation is community science. By engaging the public in data collection, the dual benefits of developing ecological literacy and furthering science can be realised (Adler et al. 2020; Grez et al. 2016; Fraisl et al. 2022). For example, in Chile, where community science allowed for the conservation status of a vulnerable trap door spider to be identified, while also educating the public on spider conservation (Barahona-Segovia et al. 2021).

## Conclusions

Here, we outlined some of the key policies for biodiversity conservation in the Neotropics. Many policies assume that they will “indirectly” conserve insect populations, either by conserving larger vertebrate species with wide home ranges or by broad habitat conservation measures. This is concerning, given that insect conservation differs from general biodiversity conservation in several ways. For example, insect conservation operates with less spatial and temporal data than many vertebrate conservation efforts, presenting challenges in identifying the conservation status of insects, and thus the true efficacy of conservation actions (Cardoso et al. 2011b; Eisenhauer et al. 2019). Insects are also smaller and less conspicuous than vertebrates, sometimes requiring a high level of taxonomic expertise to distinguish species from each other, and expertise is lacking in areas of the Neotropics for hyperdiverse groups (Brown 2005; New and Samways 2014). Finally, a lack of appreciation for insects creates challenges in building motivation for their conservation (Cardoso et al. 2011b; Sumner et al. 2018; Samways et al. 2020). In conjunction with gaps in critical policies (e.g. pesticide regulation), these challenges can potentially leave insect populations vulnerable to extinction.

**Fig. 1:**
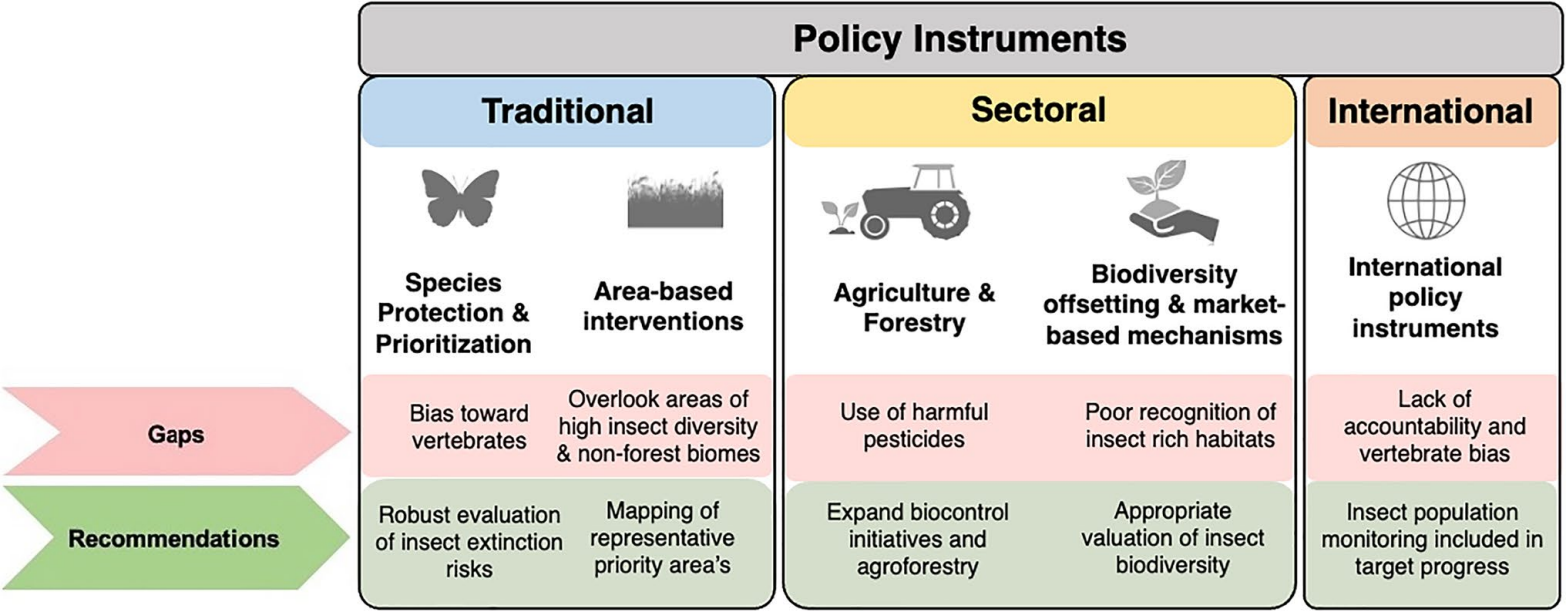
Policies affecting Neotropical biodiversity conservation, their gaps for the conservation of insects, and our recommendations to fill these policy gaps for the benefit of insect biodiversity conservation

We outlined several recommendations specific to insects summarised in Fig. 1, which will aid in delivering policies that better incorporate the conservation needs of insects: firstly, the development of more insect species-specific action policies which set out explicit goals for the conservation and further research of threatened insect groups, for example as in the recently developed Plano de Ação Nacional para a Conservação dos Insetos Polinizadores in Brazil. These initiatives should extend beyond just pollinating insects, however, in order to promote the data collection and conservation of less studied groups of insects. Such data would also aid in the designation of area-based interventions to ensure they encapsulate areas of high insect biodiversity. Additionally, further work should ensure that all ecoregions are represented in these networks, to protect the Neotropical insect endemism. This includes better representation of non-forest biomes, such as temperate grasslands, deserts and xeric shrublands. This leads to our second recommendation that working with IPLCs in ICCAs could lead to insect species persistence, through the utilisation of TEK, and the recognition of the conservation efficacy of ICCAs. Thirdly, sectors such as forestry and agriculture can provide insect habitat on the landscape scale by adopting agroecological systems and low-intensity logging, but the strengthening of pesticide regulations is urgently required. In addition, the incorporation of insect monitoring as a facet of eco-certification standards would allow for the evaluation of species recovery. However, this also extends to other sectors that impact insects, including mining, infrastructure and even tourism (Noriega et al. 2020; Silva et al. 2020; Pachecho et al. 2021). Fourthly, appropriate recognition of the non-fungible value of insect biodiversity in offsetting and restoration schemes, and protection of insect-critical habitats by PES strategies also holds excellent conservation potential. Finally, international policy instruments have a pivotal role to play in coordinating conservation efforts on the global level, but require measurable targets for the conservation of insect populations. Importantly, these recommendations are underpinned by increasing awareness of insect conservation needs and strengthening the governance of biodiversity conservation policies.

## Data Availability

This paper does not present new data.
